# Aryl Hydrocarbon Receptor Antagonists Mitigate the Effects of Dioxin on Critical Cellular Functions in Differentiating Human Osteoblast-Like Cells

**DOI:** 10.3390/ijms19010225

**Published:** 2018-01-11

**Authors:** Chawon Yun, Karina M. Katchko, Michael S. Schallmo, Soyeon Jeong, Jonghwa Yun, Charlotte H. Chen, Joseph A. Weiner, Christian Park, Andrew George, Samuel I. Stupp, Wellington K. Hsu, Erin L. Hsu

**Affiliations:** 1 Department of Orthopaedic Surgery, Northwestern University Feinberg School of Medicine, Chicago, IL 60611, USA; yunchawon@hotmail.com (C.Y.); kmkatchko@gmail.com (K.M.K.); mschallmo@hotmail.com (M.S.S.); Sophiajeong91@gmail.com (S.J.); Jonghwayun2020@u.northwestern.edu (J.Y.); jweiner07@gmail.com (J.A.W.); christiansjpark@gmail.com (C.P.); Andrew.george@northwestern.edu (A.G.); whsu@nmff.org (W.K.H.); 2 Simpson Querrey Institute for BioNanotechnology, Northwestern University, Chicago, IL 60611, USA; charlotte.h.chen@gmail.com (C.H.C.); s-stupp@northwestern.edu (S.I.S.); 3 Department of Materials Science and Engineering, Northwestern University, Evanston, IL 60208, USA; 4 Department of Biomedical Engineering, Northwestern University, Evanston, IL 60208, USA; 5 Department of Chemistry, Northwestern University, Evanston, IL 60208, USA; 6 Department of Medicine, Northwestern University, Chicago, IL 60611, USA

**Keywords:** TCDD, dioxin, bone healing, smoking, aryl hydrocarbon receptor

## Abstract

The inhibition of bone healing in humans is a well-established effect associated with cigarette smoking, but the underlying mechanisms are still unclear. Recent work using animal cell lines have implicated the aryl hydrocarbon receptor (AhR) as a mediator of the anti-osteogenic effects of cigarette smoke, but the complexity of cigarette smoke mixtures makes understanding the mechanisms of action a major challenge. 2,3,7,8-Tetrachlorodibenzo-*p*-dioxin (TCDD, dioxin) is a high-affinity AhR ligand that is frequently used to investigate biological processes impacted by AhR activation. Since there are dozens of AhR ligands present in cigarette smoke, we utilized dioxin as a prototype ligand to activate the receptor and explore its effects on pro-osteogenic biomarkers and other factors critical to osteogenesis using a human osteoblast-like cell line. We also explored the capacity for AhR antagonists to protect against dioxin action in this context. We found dioxin to inhibit osteogenic differentiation, whereas co-treatment with various AhR antagonists protected against dioxin action. Dioxin also negatively impacted cell adhesion with a corresponding reduction in the expression of integrin and cadherin proteins, which are known to be involved in this process. Similarly, the dioxin-mediated inhibition of cell migration correlated with reduced expression of the chemokine receptor *CXCR4* and its ligand, *CXCL12*, and co-treatment with antagonists restored migratory capacity. Our results suggest that AhR activation may play a role in the bone regenerative response in humans exposed to AhR activators, such as those present in cigarette smoke. Given the similarity of our results using a human cell line to previous work done in murine cells, animal models may yield data relevant to the human setting. In addition, the AhR may represent a potential therapeutic target for orthopedic patients who smoke cigarettes, or those who are exposed to secondhand smoke or other environmental sources of aryl hydrocarbons.

## 1. Introduction

The influence of tobacco smoke on human health remains an important problem worldwide. Cigarette smoke (CS) has a well-established role in the pathogenesis of numerous smoking-related disorders, including chronic obstructive pulmonary disease (COPD), lung cancer, and atherosclerosis [1,2]. However, smoking also advances musculoskeletal disease and significantly impacts the treatment of orthopedic conditions [3]. In addition to promoting osteoporosis, degenerative disc disease, and wound complications, smoking significantly inhibits osseointegration and bony union [4], which are associated with higher rates of revision procedures [5]. Smoking has also been shown to have a negative impact on outcomes after spine surgery, with a nonunion rate nearly double that of non-smokers (14.2% vs. 26.5%) [6].

Because CS contains more than 4000 distinct chemical constituents, determining a singular mechanism by which cigarette smoke inhibits bone growth is impossible [7]. Nicotine—which is present in the tar phase of cigarette smoke—has been suggested as a major mediator but its effects on osteoblasts and bone healing are nuanced; high concentrations of nicotine have been shown to inhibit osteoblast proliferation, whereas low concentrations may actually have a proliferative effect [8]. Recently, a number of studies have suggested that the aryl hydrocarbon receptor (AhR) may be a mediator of anti-osteogenic effects. The AhR is a ligand-activated basic helix-loop-helix-Per-Arnt-Sim (bHLH-PAS) family transcription factor. The receptor binds numerous exogenous ligands, as well as environmental contaminants. Dozens of the toxic constituents of CS are ligand activators of the AhR [9]. Dioxins are structurally related compounds persistent in the environment, and all of these compounds are AhR activators. 2,3,7,8-Tetrachlorodibenzo-*p*-dioxin (TCDD, referred herein as “dioxin”) has the highest affinity for the AhR and has been shown to adversely affect bone [10,11]. Downstream effects of AhR activation by ligands such as dioxin include inhibition of osteoblast differentiation, function, and reduced ossification [12,13].

Dioxin is refractory to metabolism in humans, having an estimated half-life of seven to eleven years [14]. Dioxin is also resistant to metabolism in rodents, but the half-life is in the order of weeks. This is likely due to species-specific and species-divergent responses in gene expression [15], differences in fat depots for storage [16] and elimination rates of the dioxin parent compound [15,16]. Given that differences in the half-life of dioxin between rodents and humans will lead to different effects on AhR activation and osteogenic activity, understanding the effects of dioxin and dioxin-like compounds on humans requires that human cells and tissues be used [17]. Moreover, the potential for the AhR to serve as a therapeutic target should be explored in that same context. Several AhR antagonists, some of which are found naturally in foods or are available over-the-counter as dietary supplements, have the potential to mitigate dioxin action. 3,3′-Diindolylmethane (DIM), a natural breakdown product of indole-3-carbinol, which is found in cruciferous vegetables, is a selective aryl hydrocarbon receptor modulator (SAhRM). This compound competitively binds and activates the AhR, but has distinct downstream effects from dioxin [18]. α-Napthoflavone (ANF) is also a weak AhR agonist that exhibits partial antagonist activity by competitive binding and has exhibited anti-tumorigenic activity [19]. Resveratrol, an antioxidant and antifungal present at high levels in grapes and red wine, is also a relatively potent competitive antagonist of the AhR [20]. 

Previously, we found that chronic exposure to dioxin inhibited bone morphogenetic protein 2 (BMP-2) mediated bone regeneration and posterolateral (L4–L5) spine fusion in the rat [21]. This study was the first to suggest a link between AhR activation and the reduced healing rates seen in smokers after spinal arthrodesis. Here, we sought to clarify the downstream anti-osteogenic effects of dioxin in a human osteoblast-like cell line [22,23] under the hypotheses that dioxin exposure modulates pro-osteogenic biomarkers and mediators, and that factors such as pre-osteoblast adhesion, migratory capacity, and mineral deposition would be impaired in these cells. We also explore the downstream mechanisms by which AhR activation modulates these factors. Finally, we quantify the potential for AhR antagonists to mitigate those effects, with the ultimate goal of identifying a therapeutic approach to prevent the adverse effects of cigarette smoke and other environmental sources of AhR activators on bone healing.

## 2. Results

### 2.1. Dioxin Exposure and AhR Activation

**Figure 1:**
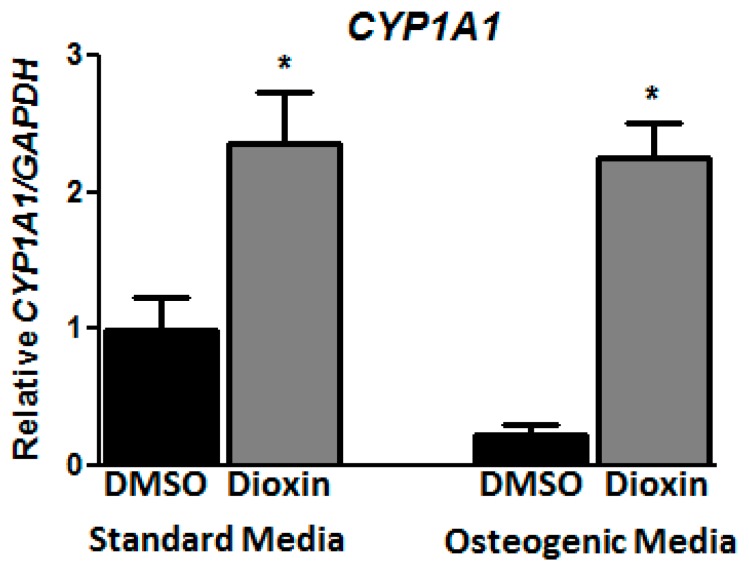
Dioxin induces *CYP1A1* under both standard and osteogenic conditions. Expression of *CYP1A1* mRNA after treatment with 100 nM dioxin or dimethyl sulfoxide (DMSO) vehicle control in cells grown under standard growth medium or osteogenic induction medium confirms that the AhR pathway is intact and functional in MG-63 cells. mRNA expression levels were normalized to DMSO vehicle control-treated cells grown in standard media. * *p* < 0.05 relative to 0 nM dioxin under standard or osteogenic conditions. Error bar means ± SEM.

At a dose of 100 nM, dioxin exposure did not change the overall cell number in standard or osteogenic conditions for the time periods evaluated in this study; this dose was chosen for subsequent differentiation experiments. Cells were analyzed for *CYP1A1* expression as a marker of dioxin exposure and subsequent AhR activation. Under standard conditions, mRNA expression for the AhR-dependent *CYP1A1* gene increased 2.36-fold after treatment with dioxin relative to dimethyl sulfoxide (DMSO)-treated cells (*p* < 0.05, Figure 1). Under osteogenic conditions, dioxin induced *CYP1A1* mRNA 8.72-fold over vehicle control-treated cells.

### 2.2. Early Markers of Osteogenic Differentiation

**Figure 2:**
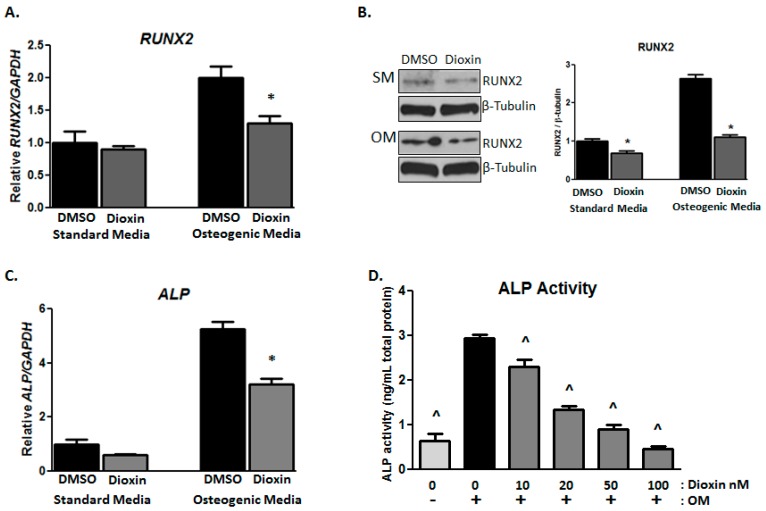
Dioxin inhibits early markers of osteogenic differentiation. (**A**) Under osteogenic conditions, dioxin significantly decreased *RUNX2* mRNA expression; (**B**) Dioxin downregulated RUNX2 protein expression under both standard and osteogenic conditions; (**C**,**D**) Dioxin significantly inhibited the osteogenic media (OM)-induced expression of *ALP* mRNA. Similarly, dioxin dose-dependently inhibited ALP activity in differentiating cells. * *p* < 0.05 relative to 0 nM dioxin under standard or osteogenic conditions (**A**–**C**); ^ *p* < 0.05 relative to 0 nM dioxin under osteogenic conditions (**D**). Error bar means ± SEM.

As expected, *RUNX2* expression in MG-63 cells was upregulated under osteogenic conditions relative to standard conditions. The mRNA was decreased by 0.79-fold and the protein level was decreased by 1.55-fold (Figure 2A,B) in cells treated with 100 nM dioxin relative to control-treated cells (*p* < 0.05). Alkaline phosphatase (*ALP*) gene expression was significantly increased under osteogenic conditions relative to standard conditions, but dioxin treatment inhibited the induction of *ALP* mRNA relative to DMSO-treated control cells (6.0- vs. 3.5-fold induction over standard media DMSO control; *p* < 0.05; Figure 2C). Similarly, ALP enzymatic activity was significantly increased in osteogenic media (OM) relative to standard media, as expected (*p* < 0.05, Figure 2D). Dioxin significantly inhibited osteogenic media-induced ALP activity at all doses tested. This effect was dose-dependent, with a significant reduction from 10 nM dioxin.

### 2.3. Cell Adhesion

**Figure 3:**
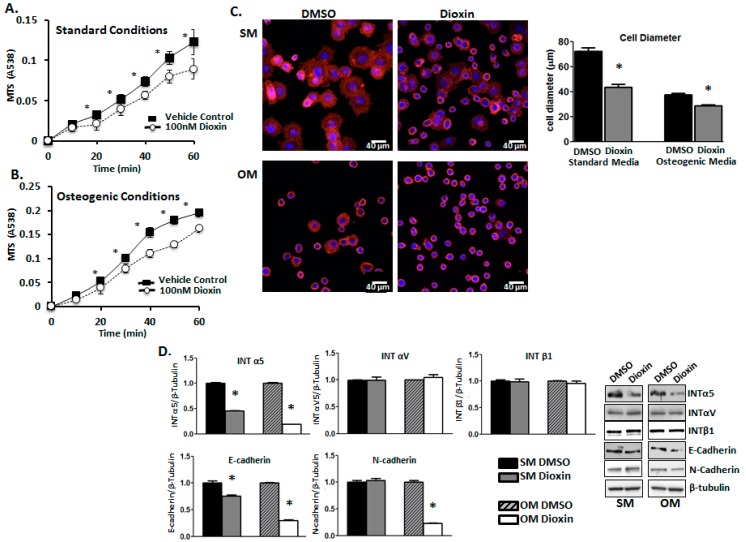
Dioxin reduces cell adhesion in both un-induced and differentiating MG-63 cells. Cell adhesion rates were quantified after a dioxin pre-treatment period of 3 days under either standard (**A**) or osteogenic conditions (**B**). Significance is shown relative to vehicle control-treated cells under both standard and osteogenic conditions; (**C**) Visualization of cell morphology. Dioxin exposure significantly decreased the proportion of flattened cells under both standard and osteogenic conditions, whereas the proportion of rounded cells was increased in response to dioxin treatment. Rhodamine-bound F-actin is shown in red, whereas nuclei are shown in blue; (**D**) Integrin (INT) α5 and E-cadherin protein expression levels were significantly decreased in dioxin-exposed cells under both standard and osteogenic conditions, whereas INTαV and INTβ1 were unchanged. N-cadherin levels were decreased only in differentiating dioxin-treated cells. * *p* < 0.05 relative to 0 nM dioxin under standard or osteogenic conditions. Error bar means ± SEM.

Cell adhesion rates were quantified using a modified MTS ((3-(4,5-dimethylthiazol-2-yl)-5-(3-carboxymethoxyphenyl)-2-(4-sulfophenyl)-2H-tetrazolium)) assay. MG-63 cells grown in the presence of dioxin under both standard (Figure 3A) and osteogenic (Figure 3B) conditions showed significantly reduced adhesion at nearly all time points relative to vehicle control-treated cells (*p* < 0.05). Cell adhesion was also visualized and quantified by measuring average cell diameter after rhodamine-conjugated phalloidin staining (Figure 3C). The average diameter of the cells was significantly lower in dioxin-treated cultures grown in both standard (72 µm vs. 41 µm) and osteogenic media conditions (38 µm vs. 32 µm), relative to respective controls (*p* < 0.05).

Integrins and cadherins have been shown to play an important role in the control of osteogenesis and osteogenic differentiation [24]. Indeed, we found that dioxin exposure affected the expression of integrin and cadherin proteins that have important functions in cell–extracellular matrix interactions (*p* < 0.05, Figure 3D). Dioxin significantly downregulated expression of integrin α5 under both standard and osteogenic conditions (1.00 vs. 0.45 and 1.0 vs. 0.22 relative expression levels, respectively) and E-cadherin protein expression (1.00 vs. 0.74 and 1.00 vs. 0.23, respectively), whereas integrin αV and integrin β1 levels were unchanged. Interestingly, N-cadherin expression was decreased by 77% in dioxin-treated cells cultured under osteogenic conditions (1.00 vs. 0.23 relative expression levels).

### 2.4. Cell Migration

**Figure 4:**
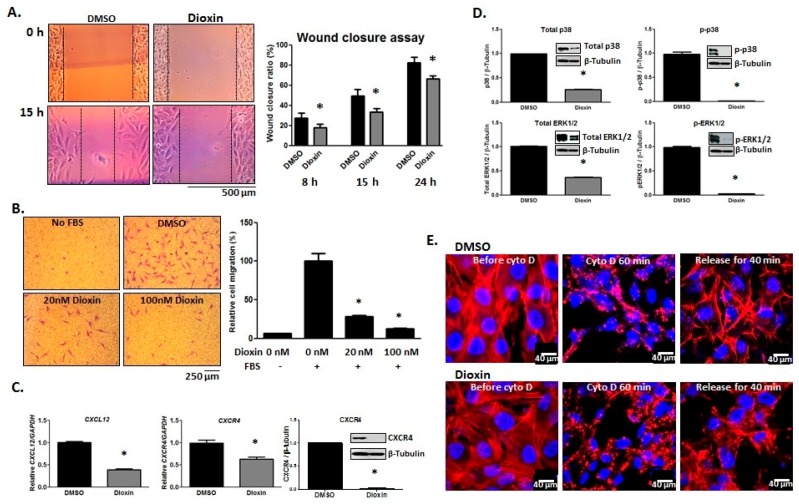
Dioxin inhibits the migratory capacity of MG-63 cells. (**A**) Dioxin-treated cells showed reduced migration across the wound space relative to DMSO-treated cells after 15 h; (**B**) Cell migration towards an FBS gradient was assessed in transwell assays. The presence of FBS in the lower chamber significantly increased the migration rate of DMSO-treated cells, whereas dioxin dose-dependently inhibited cell migration. Representative images of cells following migration towards an FBS gradient are shown; (**C**) mRNA expression levels of both *CXCL12* and its receptor, *CXCR4*, were decreased in dioxin-treated cells, and *CXCR4* protein expression was also decreased following dioxin exposure; (**D**) Dioxin significantly decreased the protein expression of total p38, active (phosphorylated) p38 (p–p38), total ERK1/2, and active (phosphorylated) ERK1/2 (p–ERK1/2). * *p* < 0.05 relative to 0 nM dioxin under standard or osteogenic conditions (**A**–**D**); (**E**) Dioxin inhibits cell adhesion/spreading, which was visualized by actin filament staining 40 min after release from cytochalasin D (cyto D) treatment. Rhodamine-bound F-actin is shown in red whereas nuclei are shown blue. Error bar means ± SEM.

The effect of dioxin exposure on the migratory capacity of MG-63 cells was assessed via wound healing and transwell chamber migration assays. Dioxin-treated cells showed a reduced capacity for migration across the “wound” space relative to DMSO-treated cells after 15 h (82% vs. 65%, respectively; *p* < 0.05, Figure 4A). In directional migration assays, the presence of FBS in the lower chamber significantly induced migration in control-treated cells as expected. However, dioxin pre-treatment caused a significant dose-dependent reduction in directional migratory capacity (at 20 nM and 100 nM dioxin) relative to control-treated cells (100% vs. 30% and 100% vs. 15%, respectively; *p* < 0.05, Figure 4B).

*CXCL12* (SDF-1) and its receptor, *CXCR4*, are known to play an important role in bone formation by influencing BMP-2 signaling [25]. Interestingly, *CXCL12* levels are increased at the site of injury and are thought to mediate the honing of progenitor cells to sites of bony injury [26,27]. We hypothesized that dioxin-treated cells, which have reduced migratory capacity, may express reduced levels of *CXCL12* and *CXCR4* relative to DMSO-treated controls. Indeed, mRNA expression of *CXCL12* was decreased in dioxin-treated cells (1.00 vs. 0.42 relative expression levels). Similarly, both mRNA and protein expression of *CXCR4* were decreased after dioxin exposure (1.00 vs. 0.64 and 1.00 vs. 0.03 relative expression levels, respectively; *p* < 0.05, Figure 4C).

Since mitogen-activated protein kinases (MAPKs) play a crucial role in cell migration [28], we also quantified the expression levels of total and active p38 and ERK1/2. Dioxin treatment significantly decreased the expression of total and active (p–p38) p38 (1.00 vs. 0.25 and 1.00 vs. 0.03, respectively), as well as total and active (p–ERK1/2) ERK1/2 (1.00 vs. 0.42 and 1.00 vs. 0.05, respectively; *p* < 0.05, Figure 4D).

Rapid actin polymerization is important for cell motility and is necessary to push forward the leading edge of migrating cells [29]. We, therefore, hypothesized that reduced cell migration after dioxin exposure may be in part due to a decrease in the rate of actin polymerization. To test this hypothesis, cells were pre-treated with DMSO or dioxin for 3 days and followed by treatment with actin de-polymerization reagent, cytochalasin D (cyto D), for 60 min. After release from cyto D for 40 min, both DMSO- and dioxin-treated cells were alyzed using Rhodamine-conjugated phalloidin.

Cells showed visible re-polymerization. However, cells pre-treated with dioxin qualitatively showed reduced organization of actin filaments (Figure 4E).

### 2.5. Matrix Mineralization

**Figure 5:**
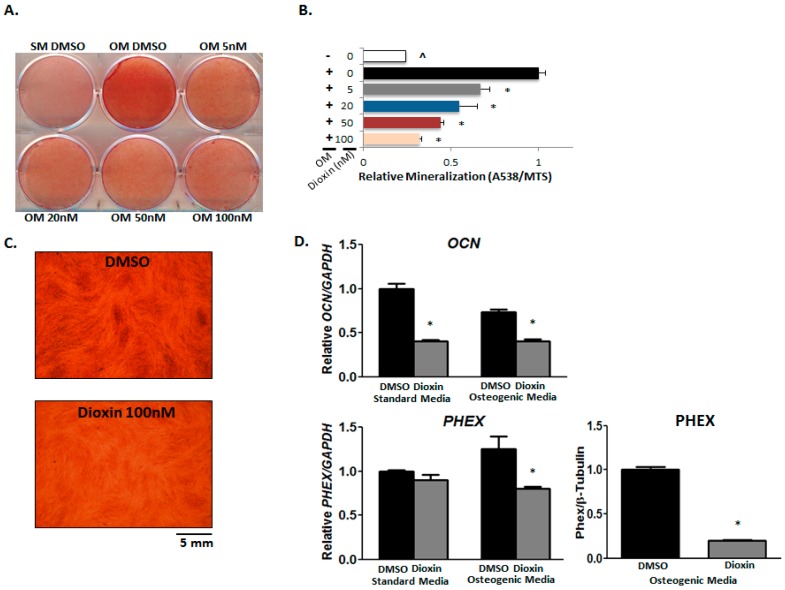
Dioxin inhibits MG-63 matrix mineralization. (**A**,**B**) Calcium deposition in the mineralized matrix was decreased by dioxin treatment. Alizarin red staining shows a dose-dependent decrease in mineralization after dioxin treatment. * *p* < 0.05, significance relative to 0 nM dioxin under osteogenic conditions; (**C**) von Kossa staining is showing that dioxin inhibits mineralization; (**D**) Osteocalcin (*OCN*) mRNA expression was significantly decreased by dioxin in cells grown under both standard and osteogenic conditions. *PHEX* mRNA expression trended downward in dioxin-treated cells under osteogenic conditions, although the difference was not significant. PHEX protein expression was significantly decreased in dioxin-treated cells. * *p* < 0.05 relative to 0 nM dioxin under standard or osteogenic conditions. Error bar means ± SEM.

Matrix mineralization by MG-63 cells was visualized using both alizarin red and von Kossa staining. Cells were treated with increasing concentrations of dioxin under osteogenic conditions. For vehicle control-treated cells, the introduction of an osteogenic environment resulted in significantly elevated mineralization relative to cells grown in standard media as expected as determined by alizarin red staining (Figure 5A).

Increasing concentrations of dioxin under osteogenic conditions dose-dependently corresponded with decreasing levels of mineral deposition with significance at all concentrations tested relative to vehicle-treated cells (*p* < 0.05, Figure 5B). The effects of dioxin on matrix mineralization were further confirmed by von Kossa staining (Figure 5C).

Factors known to influence mineralization were also impacted by dioxin exposure. Osteocalcin (*OCN*) is an osteoblast-specific marker, the expression of which has been demonstrated by staining in three-dimensional bone nodules derived from hESCs [30]. In our study, we found that dioxin decreased *OCN* mRNA both in standard and osteogenic media (1.00 vs. 0.4 and 0.78 vs. 0.38, respectively; *p* < 0.05, Figure 5D). *PHEX* is a gene that encodes for a protease responsible for the degradation and clearance of osteoblast mineralization-inhibiting acidic serine-aspartate-rich motif (ASARM) peptides as demonstrated both in vitro and in vivo [31]. Although *PHEX* mRNA expression only trended downward in dioxin-treated cells under osteogenic conditions (1.25 vs. 0.78), protein expression was significantly decreased after dioxin exposure (1.00 vs. 0.21) in osteogenic media.

### 2.6. AhR Antagonist Studies

**Figure 6:**
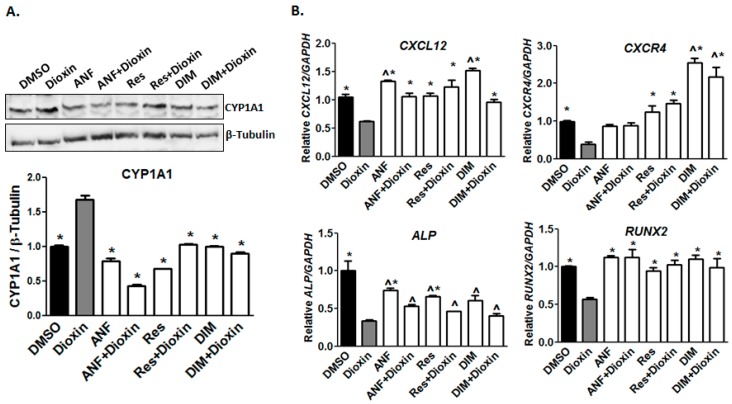
Osteogenesis-related gene expression after dioxin and AhR antagonist co-treatment. (**A**) CYP1A1 protein levels returned to near-baseline levels with AhR antagonist co-treatment. * *p* < 0.05 relative to 100 nM dioxin condition; (**B**) *CXCL12* mRNA expression was recovered by co-treatment with AhR antagonists. *CXCR4* mRNA expression was restored to control levels or higher by co-treatment with resveratrol and 3,3′-diindolylmethane (DIM). *ALP* and *RUNX2* mRNA expression was recovered by AhR antagonist co-treatment. * *p* < 0.05 relative to 100 nM dioxin condition, ^ *p* < 0.05 relative to 0 nM dioxin condition. Error bar means ± SEM.

Since the effects of dioxin on bone formation are likely mediated at least in part by the AhR [12], we hypothesized that co-treatment with dioxin and the AhR antagonists ANF, resveratrol, or DIM could prevent the dioxin-mediated inhibition of osteogenic differentiation. In preliminary work, we showed that at the relevant time points, cell proliferation was not inhibited by 0.5 µM ANF, 4 µM Res, and 10 μM DIM, and we subsequently used these doses for co-treatment studies. Notably, CYP1A1 protein was increased in dioxin-treated samples, and this induction was significantly decreased by co-treatment with AhR antagonists to near or below baseline levels (*p* < 0.05, Figure 6A). Co-treatment with ANF, Res, and DIM all significantly increased *CXCL12* mRNA. However, whereas ANF co-treatment returned *CXCR4* mRNA to normal levels (vehicle control-treated), resveratrol and DIM and co-treatment increased *CXCR4* mRNA to levels above the vehicle controls. (Figure 6B). Upon co-treatment with AhR antagonists, *ALP* levels were slightly increased over dioxin-treated samples but not fully recovered with antagonist co-treatment, whereas *RUNX2* levels were elevated relative to DMSO-treated cells.

**Figure 7:**
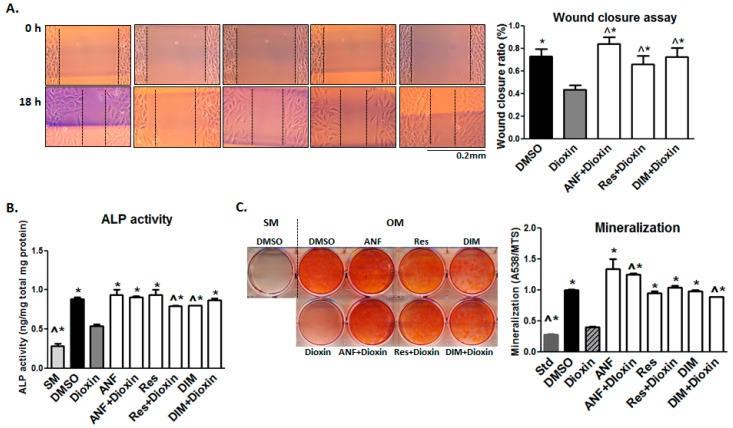
Effect of AhR antagonist co-treatment on cell migration and osteogenic differentiation. AhR antagonist co-treatment mitigated the effects of dioxin on cell migration and osteogenic differentiation. (**A**) In a wound healing chamber assay, co-treatment with ANF, resveratrol, and DIM significantly increased cell migration across a wound space compared to MG-63 cells treated with dioxin alone; (**B**,**C**) A similar mitigating effect from antagonist co-treatment was also observed for ALP activity and mineralization deposition. ^ *p* < 0.05, significance relative 0 nM dioxin (i.e., DMSO control) under osteogenic conditions, * *p* < 0.05, significance relative 100 nM dioxin under osteogenic conditions (**A**–**C**). Error bar means ± SEM.

Because dioxin inhibited the cell migration capacity (as shown in Figure 4), we investigated whether the antagonists could reduce the negative impact of dioxin on migration. In wound healing assays, co-treatment with AhR antagonists significantly increased cell migration across the wound space compared to MG-63 cells treated with dioxin alone (*p* < 0.05, Figure 7A). A similar rescue effect was recapitulated in ALP activity and mineralization assays, where each of the three antagonists tested provided a significant protective effect relative to dioxin alone (*p* < 0.05, Figure 7B,C). ANF demonstrated the strongest rescue effect on both cell migration and mineralization in dioxin-treated cells, with a full restoration to control levels; resveratrol and DIM also showed significant mitigating effects relative to dioxin-treated cells.

## 3. Discussion

Cigarette smoking greatly contributes to negative outcomes after spinal fusion procedures and rates of non-healing are significantly elevated in smokers (14% vs. 26%) [6]. Although smoking rates are on the decline, an estimated 20–25% of Americans still smoke and many more are subject to secondhand smoke [32]. These patients present a major dilemma for orthopedic surgeons who are severely limited in their ability to treat habitual smokers and who are left with the difficult choice of refusing surgical intervention or performing the procedure with significantly increased risks. A number of clinical studies have linked smoking with significant inhibitory effects on bone healing; however, inadequate clarification of the underlying molecular mechanisms has prevented the development of an appropriate therapeutic approach [33,34].

While it has been posited that a number of constituents of cigarette smoke—including nicotine—may play a role in the smoking-mediated inhibition of bone healing, the role of the aryl hydrocarbon receptor (AhR) has become a recent focus of active research [35,36]. The AhR is responsible for regulating xenobiotic metabolism and mediating the toxicity of environmental planar aromatic hydrocarbons, with previous studies suggesting deleterious effects of AhR hyper-activation on bone biology [12,13,37].

Dioxin, a minor hydrocarbon constituent of cigarette smoke but potent activator of the AhR, is thought to exert its effects almost exclusively through the receptor [20,21]. Because of its extremely high affinity for the receptor, dioxin is frequently used as a prototypical ligand to investigate the effects of the AhR pathway involvement in biological processes. Previous in vitro studies have shown that dioxin inhibits osteoblastic differentiation and ALP activity in murine cell lines and have also identified dioxin-sensitive proteins that are critical mediators of osteogenesis [12,38].

To confirm whether the AhR pathway is functional in MG-63 cells, we examined *CYP1A1* expression, a well-established marker of AhR activation. Its induction by dioxin has confirmed that the AhR pathway is indeed intact in both non-differentiating and differentiating MG-63 cells. All three AhR antagonists reduced *CYP1A1* expression to near baseline levels in dioxin-exposed cells (Figure 6), indicating that they are potent enough to achieve successful antagonism of the AhR in this setting.

Previous in vitro studies using both human and animal cell lines have explored changes in cell adhesion after dioxin treatment, as well as direct effects on integrins and cadherins in human pre-osteoblasts [38,39]. The reduced cellular adhesion rates we observed under both standard and osteogenic conditions may have implications for osteogenesis in vivo, where an inhibition of progenitor cell adhesion at the site of injury or scaffold implant could have adverse effects on the regenerative capacity (Figure 3). The observed reduction in cell adhesion rate and integrin expression levels demonstrates that dioxin may directly or indirectly modulates the expression or activity of adhesion-associated molecules, which could alter cell–cell and cell–matrix interactions. Further work should be done to determine if AhR antagonists can also mitigate the dioxin-mediated inhibition of cell adhesion and integrin expression.

*CXCL12* is predominantly expressed by osteogenic stem and precursor cells prior to and immediately following the onset of osteogenesis, but its expression rapidly declines once cells commit to differentiation. Numerous groups have shown that *CXCL12* signaling plays a role in the initiation of osteogenic differentiation. The interaction between *CXCL12* and *CXCR4* leads to both cytoskeletal rearrangement and migration of *CXCR4*-expressing cells toward a *CXCL12* gradient [40]. Corresponding with our observed decrease in *CXCL12* and *CXCR4* mRNA expression, we found that dioxin exposure resulted in a decreased capacity for cell migration (Figure 4 and Figure 6). Previous studies have shown *CXCL12* upregulation at sites of injury, where it serves to recruit *CXCR4*-expressing stem cells in support of tissue-specific repair or regeneration [41]. Clinically, a reduction in progenitor cell motility could drastically impact the capacity for regeneration, since progenitors actively migrate towards an array of chemoattractants at the site of tissue injury. We postulate that dioxin-mediated downregulation of *CXCL12* could play an important role in its adverse effects on bone regeneration in vivo, which will be explored in future work.

Notably, the expression levels of *PHEX* were significantly reduced in dioxin-treated differentiating MG-63 cells. PHEX is a zinc metalloendopeptidase that inhibits cleavage of the MEPE ASARM motif of SIBLING proteins (short integrin-binding ligand-interacting glycoproteins), which regulate mineralization. The released (free) ASARM peptide plays a major regulatory role in the mineralization of bones and teeth, as well as soft-tissue calcification, by binding strongly to hydroxyapatite and inhibiting mineralization [42]. We suspect that the downregulation of *PHEX* by dioxin—which would presumably result in an increase in levels of free ASARM—may lead to reduced mineral deposition, and this mechanism may be in part responsible for the decreased mineralization capacity and inhibition of bone healing seen in dioxin-exposed cells and animals.

With this work, we found that ALP activity is decreased after dioxin treatment but can be recovered with AhR antagonist co-treatment. The p38/ERK MAPK pathway, which is normally enhanced by BMP-2, plays an important role in osteoblastic differentiation and mineralization [43]. We found a decrease in overall p38/ERK and active p–p38/p–ERK levels (Figure 4). This may in part explain the decreased ALP activity and mineralization in dioxin-treated cells. Whether p38 and ERK levels are recovered with AhR antagonist co-treatment will be examined in subsequent studies.

Overall, we observed that DIM (a selective AhR modulator), ANF (a partial AhR agonist/antagonist), and Res (a competitive AhR antagonist) worked similarly to promote osteogenesis in cells exposed to dioxin. Numerous AhR antagonists, such as resveratrol and DIM, can be found in an assortment of fruits and vegetables, as well as in over-the-counter dietary supplements. This class of compounds may represent a naturally sourced pharmacologic approach to combat the deleterious effects of cigarette smoke on bony healing. Bioavailability of these compounds is often low, making it difficult to achieve therapeutic concentrations with standard formulations currently on the market. Development of sustained-release formulations should be pursued to determine whether they can provide clinically significant protective effects.

Although our study delineates anti-osteogenic effects specific to dioxin, there are dozens of other ligand activators of the AhR present in cigarette smoke [9,43]. As such, these chemicals may have similar and potentially cumulative inhibitory effects on osteogenesis. The stability of these compounds varies drastically. For example, the half-life of dioxin is estimated to be 7 to 11 years, while the half-life of the polycyclic aromatic hydrocarbon, benzo[a]pyrene, is much shorter [14]. This may lead to variation in the impact that individual compounds have on the AhR and in turn bone healing. Identification of non-toxic compounds with antagonistic but not agonistic effects on the AhR is potentially of great medical interest and could improve the surgical outcomes of patients who smoke or have environmental or dietary exposure to AhR agonists.

Prior studies have focused on the isolated effects and mechanisms of specific chemical constituents present in cigarette smoke, but the cumulative effects of whole smoke on AhR activation and bone regeneration are not well understood. While it is important to appreciate the inhibitory role of individual chemicals such as dioxin on bone healing, the more clinically-relevant question is how the multitude of AhR ligands present in cigarette smoke collectively impact bone regeneration. Future studies should focus on clarifying the contribution of AhR activation by cumulative exposure to these various ligands contained within cigarette smoke to their inhibitory effects on bone regeneration and healing. There is also potential for additive or synergistic effects from treatment with multiple AhR antagonists, and this should be pursued in future work.

With a limited ability to treat patients who smoke and the low compliance with surgeons’ requests for smoking cessation, an effective measure to reduce risk and improve patient outcomes would be extremely beneficial. This research identifies AhR antagonists that may provide a protective effect towards the harmful consequences of environmental contaminants present in cigarette smoke and other sources, and provides an argument for the development of therapeutics that target the AhR pathway to reduce the negative impact of cigarette smoke on bone.

## 4. Materials and Methods

### 4.1. MG-63 Cell Culture

Human MG-63 osteosarcoma cells, which are pre-osteoblast-like cells that differentiate into osteoblasts upon growth in osteoinductive media, were purchased from American Type Culture Collection (ATCC CRL-1427; Rockville, MD, USA). Cells were grown in Dulbecco’s Modified Eagle Medium (DMEM; Gibco, Carlsbad, CA, USA) containing 10% fetal bovine serum (FBS) and 1% penicillin/streptomycin solution (standard media, SM), at 37 °C in a humidified atmosphere of 5% CO_2_. For all cell culture assays, media was replaced every 2–3 days. For induction of osteogenic differentiation, DMEM was supplemented with 15 mM β-glycerophosphate, 75 ng/mL l-ascorbic acid, and 15 nM dexamethasone (osteogenic media, OM). A dose of 100 nM dioxin was selected based on previous studies revealing 100% AhR binding saturation without cytotoxicity [44]. We also evaluated the effect of each antagonist on cell proliferation and found non-cytotoxic concentrations of α-naphthoflavone (ANF), resveratrol (Res), and 3,3′-Diindolylmethane (DIM) over the time period. These doses were therefore utilized for subsequent differentiation studies. Cells were treated with either vehicle control (dimethyl sulfoxide, DMSO; 0.1% final concentration) or the following: 100 nM dioxin, 0.5 µM ANF, 4 µM Res, and 10 µM DIM. All chemicals were purchased from Sigma-Aldrich (St. Louis, MO, USA).

### 4.2. Alkaline Phosphatase Activity Assays

Cellular alkaline phosphatase (ALP) activity was quantified using a SensoLyte pNPP alkaline phosphate assay kit (Anaspec, Fermont, CA, USA). Cells were grown in standard or osteogenic media and treated with DMSO or dioxin for 7 days. Enzymatic reactions were performed on lysate supernatants as instructed by the manufacturer. A minimum of three independent experiments were performed for quantitation of ALP activity, as well as for all other in vitro assays.

### 4.3. Cell Adhesion Assays

MG-63 cells were grown under standard or osteogenic conditions and pre-treated with DMSO vehicle or 100 nM dioxin for 3 days. Then, 1.25 × 10^4^ cells were inoculated into 96-well plates pre-coated with 8 µg/mL fibronectin. At 10-min increments, non-adherent cells were removed with a wash in PBS, and the remaining adherent cells were incubated with MTS (3-(4,5-dimethylthiazol-2-yl)-5-(3-carboxymethoxyphenyl)-2-(4-sulfophenyl)-2H-tetrazolium) assay reagent (Promega, Madison, WI, USA) for indirect quantification of cell number. For confocal microscopy, DMSO- and dioxin-treated cells were inoculated onto fibronectin-coated coverslips and incubated for 60 min. Cells were fixed with 4% paraformaldehyde for 20 min, washed with PBS, and incubated with 5% bovine serum albumin (BSA) for 1 h. Cells were then stained with rhodamine-conjugated phalloidin for 1 h to visualize actin filaments, followed by staining with 4′,6-diamidino-2-phenylindole (DAPI) to visualize nuclei. Confocal images were captured using a Nikon A1R microscope (Nikon, Tokyo, Japan).

### 4.4. Actin Filament Re-Polymerization Assay

MG-63 cells were directly seeded onto 8 µg/mL fibronectin-coated coverslips and treated with DMSO or dioxin for 3 days, followed by exposure to 1 µM of cytochalasin D for 1 h to depolymerize the actin cytoskeleton. After washing with PBS, cells were incubated with media to allow for actin re-polymerization. After 40 min, cells were fixed with 4% paraformaldehyde, blocked with 5% BSA for 1 h, and stained with rhodamine-conjugated phalloidin and DAPI for visualization of actin filament re-polymerization.

### 4.5. Cell Migration Assays

For wound healing assays, cells were maintained in “Culture-Insert 3 well plates” (ibidi, Madison, WI, USA) with DMSO- or dioxin-treated media. When cells reached confluence, the inserts were removed from the wells and the wells were washed twice with PBS. Cells were then incubated in DMSO- or dioxin-containing media for 15 h. Digital images were collected at time points 0, 8, 15, and 24 h using a light microscope in order to evaluate the rate of “wound closure.” Transwell assays were performed to measure the effect of dioxin on directional cell migration capacity using previously published methods [19].

### 4.6. Mineralization Assays

Matrix mineralization was quantified using alizarin red staining followed by a cetylpyridinium chloride de-stain procedure, using previously published methodology [12]. After 3 min, cells were thoroughly washed and then de-stained with cetylpyridinium chloride. Absorbance of the de-stain solution was quantified using a Cytation 3 spectrophotometer at A_538nm_ (BioTek Instruments, Winooski, VT, USA). Mineralization was also confirmed by von Kossa staining by a previously established protocol [45].

### 4.7. Western Blotting

Rapid-immunoprecipitation assay (RIPA) buffer, blocking solution, and protease inhibitor were purchased from GenDepot (Barker, TX, USA). β-Tubulin and RUNX2 antibodies were purchased from Proteintech (Rosemont, IL, USA), and integrin α5, integrin αV, N-cadherin, E-cadherin, and integrin β1 antibodies were purchased from Santa Cruz Biotechnology (Dallas, TX, USA). Total p38, p–p38, total ERK1/2, and p–ERK1/2 antibodies were purchased from Cell Signaling Technology (Billerica, MA, USA). After primary antibody incubation, membranes were washed with PBST and incubated with horseradish conjugated secondary antibodies for 1 h at room temperature. Signal was visualized by enhanced chemiluminescence (ECL) using Azure300 (Azure Biosystems, Dublin, CA, USA), and intensities were quantified using a computing densitometry program from Image Studio Lite (LI-COR, Lincoln, NE, USA).

### 4.8. RNA Isolation and Gene Expression Analysis

Primer sets for human genes used in this study are listed in Table 1*.* Primers were synthesized by Integrated DNA Technologies (Coralville, IA, USA). Expression levels of mRNA were quantified using quantitative polymerase chain reaction (qPCR), which was performed using a Bio-Rad iQ5 Multicolor Real-Time PCR Detection System (Bio-Rad, Hercules, CA, USA) in the Equipment Core Facility of the Simpson Querrey Institute at Northwestern University. RNAsol, MinElute columns, complementary DNA (cDNA) synthesis kits, and 2X amfiSure qGreen qPCR Master Mix 2 were purchased by GenDepot (Barker, TX, USA). QPCR was performed using the following program: 94 °C denaturation for 5 min; then 40 repeated cycles of 94 °C, 45 s/55 °C, 1 min/68 °C for 1 min; 79 cycles at 55°C for 30 s each for generation of melting curves. Relative gene expression was analyzed using the Livak method [46]. A normalized expression ratio (2^−ΔΔ*C*t^) was calculated for each target gene, representing a fold difference in mRNA expression for treatment groups relative to vehicle control.

### 4.9. Statistical Analysis

Data were analyzed for overall statistical significance using a two-way ANOVA using Prism GraphPad (La Jolla, CA, USA). *p*-values less than 0.05 were regarded as statistically significant.

## Figures and Tables

**Table 1:** Primer sets for qPCR.

cDNA		Sequences 5′-3′
*CYP1A1*	Forward	AAA CCC AGC TGA CTT CAT CC
	Reverse	TGC TCC TTG ACC ATC TTC TG
*RUNX2*	Forward	GGT TAA TCT CCG CAG GTC ACT
	Reverse	CAC TGT GCT GAA GAG GCT GTT
*ALP*	Forward	CCA TTC CCA CGT CTT CAC AT
	Reverse	GCT TCT TGT CTG TGT CAC TCA
*CXCL12*	Forward	TGC CAG AGC CAA CGT CAA G
	Reverse	CAG CCG GGC TAC AAT CTG AA
*CXCR4*	Forward	AGC AGG TAG CAA AGT GAC G
	Reverse	CCT CGG TGT AGT TAT CTG AAG TG
*PHEX*	Forward	GAG CTC AAG TTA TGC TCA TGT GAG GTG
	Reverse	AAA TAA GAG CTC CAG AGT CGA CAG GAG
*OCN*	Forward	TCA CAC TCC TCG CCC TAT TG
	Reverse	TCG CTG CCC TCC TGC TTG

## References

[1] Middlekauff H.R., Park J., Moheimani R.S. (2014). Adverse effects of cigarette and noncigarette smoke exposure on the autonomic nervous system: Mechanisms and implications for cardiovascular risk. J. Am. Coll. Cardiol..

[2] Sasco A.J., Secretan M.B., Straif K. (2004). Tobacco smoking and cancer: A brief review of recent epidemiological evidence. Lung Cancer.

[3] Porter S.E., Hanley E.N. (2001). The musculoskeletal effects of smoking. J. Am. Acad. Orthop. Surg..

[4] Sloan A., Hussain I., Maqsood M., Eremin O., El-Sheemy M. (2010). The effects of smoking on fracture healing. Surgeon.

[5] Bydon M., De la Garza-Ramos R., Abt N.B., Gokaslan Z.L., Wolinsky J.P., Sciubba D.M., Bydon A., Witham T.F. (2014). Impact of smoking on complication and pseudarthrosis rates after single- and 2-level posterolateral fusion of the lumbar spine. Spine.

[6] Glassman S.D., Anagnost S.C., Parker A., Burke D., Johnson J.R., Dimar J.R. (2000). The effect of cigarette smoking and smoking cessation on spinal fusion. Spine.

[7] Hoffmann D., Djordjevic M.V., Hoffmann I. (1997). The changing cigarette. Prev. Med..

[8] Rothem D.E., Rothem L., Soudry M., Dahan A., Eliakim R. (2009). Nicotine modulates bone metabolism-associated gene expression in osteoblast cells. J. Bone Miner. Metab..

[9] Leow Y.H., Maibach H.I. (1998). Cigarette smoking, cutaneous vasculature, and tissue oxygen. Clin. Dermatol..

[10] Lee L.L., Lee J.S., Waldman S.D., Casper R.F., Grynpas M.D. (2002). Polycyclic aromatic hydrocarbons present in cigarette smoke cause bone loss in an ovariectomized rat model. Bone.

[11] Kung M.H., Yukata K., O’Keefe R.J., Zuscik M.J. (2012). Aryl hydrocarbon receptor-mediated impairment of chondrogenesis and fracture healing by cigarette smoke and benzo(a)pyrene. J. Cell. Physiol..

[12] Yun C., Weiner J.A., Chun D.S., Yun J., Cook R.W., Schallmo M.S., Kannan A.S., Mitchell S.M., Freshman R.D., Park C. (2017). Mechanistic insight into the effects of Aryl Hydrocarbon Receptor activation on osteogenic differentiation. Bone Rep..

[13] Jamsa T., Viluksela M., Tuomisto J.T., Tuomisto J., Tuukkanen J. (2001). Effects of 2,3,7,8-tetrachlorodibenzo-p-dioxin on bone in two rat strains with different aryl hydrocarbon receptor structures. J. Bone Miner. Res..

[14] Milbrath M.O., Wenger Y., Chang C.W., Emond C., Garabrant D., Gillespie B.W., Jolliet O. (2009). Apparent half-lives of dioxins, furans, and polychlorinated biphenyls as a function of age, body fat, smoking status, and breast-feeding. Environ. Health Perspect..

[15] Boutros P.C., Yan R., Moffat I.D., Pohjanvirta R., Okey A.B. (2008). Transcriptomic responses to 2,3,7,8-tetrachlorodibenzo-p-dioxin (TCDD) in liver: Comparison of rat and mouse. BMC Genom..

[16] Emond C., Birnbaum L.S., DeVito M.J. (2006). Use of a physiologically based pharmacokinetic model for rats to study the influence of body fat mass and induction of CYP1A2 on the pharmacokinetics of TCDD. Environ. Health Perspect..

[17] Byard J.L. (1987). The toxicological significance of 2,3,7,8-tetrachlorodibenzo-p-dioxin and related compounds in human adipose tissue. J. Toxicol. Environ. Health.

[18] Hestermann E.V., Brown M. (2003). Agonist and chemopreventative ligands induce differential transcriptional cofactor recruitment by aryl hydrocarbon receptor. Mol. Cell. Biol..

[19] Merchant M., Krishnan V., Safe S. (1993). Mechanism of action of alpha-naphthoflavone as an Ah receptor antagonist in MCF-7 human breast cancer cells. Toxicol. Appl. Pharmacol..

[20] Neal M.S., Mulligan Tuttle A.M., Casper R.F., Lagunov A., Foster W.G. (2010). Aryl hydrocarbon receptor antagonists attenuate the deleterious effects of benzo[a]pyrene on isolated rat follicle development. Reprod. Biomed. Online.

[21] Hsu E.L., Sonn K., Kannan A., Bellary S., Yun C., Hashmi S., Nelson J., Mendoza M., Nickoli M., Ghodasra J. (2015). Dioxin Exposure Impairs BMP-2-Mediated Spinal Fusion in a Rat Arthrodesis Model. J. Bone Jt. Surg. Am. Vol..

[22] Benayahu D., Shur I., Marom R., Meller I., Issakov J. (2001). Cellular and molecular properties associated with osteosarcoma cells. J. Cell. Biochem..

[23] Jukkola A., Risteli L., Melkko J., Risteli J. (1993). Procollagen synthesis and extracellular matrix deposition in MG-63 osteosarcoma cells. J. Bone Miner. Res..

[24] Marie P.J., Hay E., Saidak Z. (2014). Integrin and cadherin signaling in bone: Role and potential therapeutic targets. Trends Endocrinol. Metab..

[25] Gilchrist Annette S.P. (2015). Chemokines and Bone. Clin. Rev. Bone Miner. Metab..

[26] Kawakami Y., Ii M., Matsumoto T., Kuroda R., Kuroda T., Kwon S.M., Kawamoto A., Akimaru H., Mifune Y., Shoji T. (2015). SDF-1/CXCR4 axis in Tie2-lineage cells including endothelial progenitor cells contributes to bone fracture healing. J. Bone Miner. Res..

[27] Yellowley C. (2013). CXCL12/CXCR4 signaling and other recruitment and homing pathways in fracture repair. Bonekey Rep..

[28] Huang C., Jacobson K., Schaller M.D. (2004). MAP kinases and cell migration. J. Cell Sci..

[29] Wang Y.L. (1985). Exchange of actin subunits at the leading edge of living fibroblasts: Possible role of treadmilling. J. Cell Biol..

[30] Bonewald L.F., Harris S.E., Rosser J., Dallas M.R., Dallas S.L., Camacho N.P., Boyan B., Boskey A. (2003). von Kossa staining alone is not sufficient to confirm that mineralization in vitro represents bone formation. Calcif. Tissue Int..

[31] Addison W.N., Masica D.L., Gray J.J., McKee M.D. (2010). Phosphorylation-dependent inhibition of mineralization by osteopontin ASARM peptides is regulated by PHEX cleavage. J. Bone Miner. Res..

[32] Hadley M.N., Reddy S.V. (1997). Smoking and the human vertebral column: A review of the impact of cigarette use on vertebral bone metabolism and spinal fusion. Neurosurgery.

[33] Hernigou J., Schuind F. (2013). Smoking as a predictor of negative outcome in diaphyseal fracture healing. Int. Orthop..

[34] Hilibrand A.S., Fye M.A., Emery S.E., Palumbo M.A., Bohlman H.H. (2001). Impact of smoking on the outcome of anterior cervical arthrodesis with interbody or strut-grafting. J. Bone Jt. Surg. Am. Vol..

[35] Lind P.M., Wejheden C., Lundberg R., Alvarez-Lloret P., Hermsen S.A., Rodriguez-Navarro A.B., Larsson S., Rannug A. (2009). Short-term exposure to dioxin impairs bone tissue in male rats. Chemosphere.

[36] Korkalainen M., Kallio E., Olkku A., Nelo K., Ilvesaro J., Tuukkanen J., Mahonen A., Viluksela M. (2009). Dioxins interfere with differentiation of osteoblasts and osteoclasts. Bone.

[37] Carpi D., Korkalainen M., Airoldi L., Fanelli R., Hakansson H., Muhonen V., Tuukkanen J., Viluksela M., Pastorelli R. (2009). Dioxin-sensitive proteins in differentiating osteoblasts: Effects on bone formation in vitro. Toxicol. Sci..

[38] Guo H., Zhang L., Wei K., Zhao J., Wang Y., Jin F., Xuan K. (2015). Exposure to a continuous low dose of tetrachlorodibenzo-p-dioxin impairs the development of the tooth root in lactational rats and alters the function of apical papilla-derived stem cells. Arch. Oral Biol..

[39] Rowe P.S. (2012). Regulation of bone-renal mineral and energy metabolism: The PHEX, FGF23, DMP1, MEPE ASARM pathway. Crit. Rev. Eukaryot. Gene Expr..

[40] Rowe P.S., Garrett I.R., Schwarz P.M., Carnes D.L., Lafer E.M., Mundy G.R., Gutierrez G.E. (2005). Surface plasmon resonance (SPR) confirms that MEPE binds to PHEX via the MEPE-ASARM motif: A model for impaired mineralization in X-linked rickets (HYP). Bone.

[41] Burger J.A., Kipps T.J. (2006). CXCR4: A key receptor in the crosstalk between tumor cells and their microenvironment. Blood.

[42] Kitamura M., Kasai A. (2007). Cigarette smoke as a trigger for the dioxin receptor-mediated signaling pathway. Cancer Lett..

[43] Singh S., Singh U.P., Grizzle W.E., Lillard J.W. (2004). CXCL12-CXCR4 interactions modulate prostate cancer cell migration, metalloproteinase expression and invasion. Lab. Investig..

[44] Connor K.T., Aylward L.L. (2006). Human response to dioxin: Aryl hydrocarbon receptor (AhR) molecular structure, function, and dose-response data for enzyme induction indicate an impaired human AhR. J. Toxicol. Environ. Health Part B Crit. Rev..

[45] Wang Y.H., Liu Y., Maye P., Rowe D.W. (2006). Examination of mineralized nodule formation in living osteoblastic cultures using fluorescent dyes. Biotechnol. Prog..

[46] Livak K.J., Schmittgen T.D. (2001). Analysis of relative gene expression data using real-time quantitative PCR and the 2(-Delta Delta C(T)) Method. Methods.

